# Identification of metal ion binding sites based on amino acid sequences

**DOI:** 10.1371/journal.pone.0183756

**Published:** 2017-08-30

**Authors:** Xiaoyong Cao, Xiuzhen Hu, Xiaojin Zhang, Sujuan Gao, Changjiang Ding, Yonge Feng, Weihua Bao

**Affiliations:** 1 College of Sciences, Inner Mongolia University of Technology, Hohhot, 010051, China; 2 College of Sciences, Inner Mongolia Agricultural University, Hohhot, 010021, China; Russian Academy of Medical Sciences, RUSSIAN FEDERATION

## Abstract

The identification of metal ion binding sites is important for protein function annotation and the design of new drug molecules. This study presents an effective method of analyzing and identifying the binding residues of metal ions based solely on sequence information. Ten metal ions were extracted from the BioLip database: Zn^2+^, Cu^2+^, Fe^2+^, Fe^3+^, Ca^2+^, Mg^2+^, Mn^2+^, Na^+^, K^+^ and Co^2+^. The analysis showed that Zn^2+^, Cu^2+^, Fe^2+^, Fe^3+^, and Co^2+^ were sensitive to the conservation of amino acids at binding sites, and promising results can be achieved using the Position Weight Scoring Matrix algorithm, with an accuracy of over 79.9% and a Matthews correlation coefficient of over 0.6. The binding sites of other metals can also be accurately identified using the Support Vector Machine algorithm with multifeature parameters as input. In addition, we found that Ca^2+^ was insensitive to hydrophobicity and hydrophilicity information and Mn^2+^ was insensitive to polarization charge information. An online server was constructed based on the framework of the proposed method and is freely available at http://60.31.198.140:8081/metal/HomePage/HomePage.html.

## Introduction

Approximately one-third of all known proteins bind with metal ions [1,2]. The metal ions play a crucial role in protein structure and function, for example the transportation of iron ions in hemoglobin, the stabilization of zinc ions in the zinc finger domain, and the regulation of calcium ions in calmodulin [3–7]. The realization of biological function depends on the interaction between the ligand-binding residues and metal ions. The molecular mechanism involves the metal ions binding with specific residues within proteins. In addition, the role of metal ions in dSPNs [8,9] (disease-related single nucleotide polymorphisms) is directly related to human disease, and the identification of metal ion-binding residues is of great significance for the development of molecular drugs to treat human diseases.

During the last few years, many approaches have been developed to predict the binding sites of protein-metal ions. The methods of identifying metal ion-binding residues are generally divided into two types. One type of method directly predicts the metal ion binding sites using 3D structural information, and high accuracy can be achieved. The Fold-X force field algorithm was used by Joost et al. [10] to predict Ca^2+^, Zn^2+^, Cu^2+^ and Mn^2+^ ion binding residues, obtaining an overall accuracy from 90% to 97%. Deng et al. [11] developed graph theory-based and geometry-based approaches to detecting calcium-binding sites and achieved a sensitivity of nearly 90% for 123 calcium binding proteins. The CHED algorithm was developed by Babor et al. [12,13] based on the three-dimensional (3D) structure to predict transition metal-binding sites (Zn^2+^, Co^2+^, Ni^2+^, Fe^2+^, Cu^2+^, and Mn^2+^) in 349 apoproteins and 82 holoproteins, achieving specificities of 95% and 96%, respectively. Jessica et al. [14] developed a Bayesian classifier to predict zinc-binding sites in 349 zinc proteins and achieved a specificity of 99.8% and sensitivity of 75.5%. Yang et al. [15] constructed the online server I-TASSER suite based on sequence and structure information and predicted the ligand binding sites of proteins by integrating many algorithms, including TM-SITE [16] and COFACTER [17], in series. The method was evaluated in CASP11 [18] and performed very well.

For most proteins, the 3D structure has not been derived. The alternative methods use the amino acid sequence information to identify the binding residues of metal ions in proteins, and although the prediction accuracy is generally lower, this method is more universal. The Metsite approach was developed by JS Sodhi et al. [19] using artificial neural networks to predict the binding sites of six metal ions (Ca^2+^, Cu^2+^, Mg^2+^, Fe^3+^, Mn^2+^ and Zn^2+^) on 1018 protein chains. The method achieved an accuracy of 94.5% by 5-fold cross-validation. In 2005, Lin et al. [20] predicted the protein metal-binding residues from sequence information using artificial neural networks; the method yielded a sensitivity higher than 90% and was very accurate under 5-fold cross-validation. In 2006, Lin et al. [21] used SVM prediction systems that were trained on a dataset containing 53,333 metal-binding residues to predict the binding residues of ten metal ions. The method was evaluated on an independent set of 31,448 metal-binding residues, and the computed prediction accuracy was higher than 74.9%. Lu et al. [22] predicted the metal ion-binding sites (Ca^2+^, Mg^2+^, Cu^2+^, Fe^3+^, Mn^2+^ and Zn^2+^) in proteins by the fragment transformation method using both sequence and structural information and achieved an overall accuracy of 94.6% with a true positive rate of 60.5%. Hu et al. [23] developed a composite method (IonCom) that combines the ab initio model with multiple threading alignments for 9 metal ion binding site predictions and observed good results under 5-fold cross-validation.

The study of the binding residues of multiple metal ion ligands generally uses the same characteristic parameters and the same prediction model. In fact, each metal ion ligand binding residue is different, and no single characteristic parameter can be sensitive to all metal ligands; this is the reason for the different results. We aim to predict metal ion binding sites based on only sequence information and to obtain robust results. In this study, based on sequence information, the binding residues of 10 kinds of metal ions were derived using statistical analysis and a prediction algorithm. At the same time, the sensitive characteristics of different types of metal ion binding residues were derived by calculation, and the proposed prediction algorithms were evaluated by cross-validation and independent tests. This approach also utilized a position-weighted scoring matrix and a support vector machine learning algorithm to evaluate data and refine predictions. This combination of methods and analytical approaches has culminated in a relatively effective tool for predicting metal binding sites without the use of 3D structures. The advantages and disadvantages of our method are discussed.

## Materials and methods

### Non-redundant dataset

The proteins interacting with metal ions were downloaded from the BioLiP [24] database using a pairwise sequence identity below 95%. There are ten metal ions that have a sufficient number of binding residues to perform the statistical analysis, i.e., Zn^2+^, Cu^2+^, Fe^2+^, Fe^3+^, Ca^2+^, Mg^2+^, Mn^2+^, Na^+^, K^+^ and Co^2+^. The proteins were further filtered by keeping only those with a resolution less than 3.0 Å and a sequence length greater than 50 residues. Redundant proteins were removed using the CD-HIT program [25] with a sequence identity threshold of 30%. Table 1 shows the summary statistics of the dataset. The number of protein chains varied from 57 to 1428 for different metal ions. The binding segment was defined as the sequence segment with the binding residue centered in a fixed-length window. A similar definition was used to specify the non-binding segments, where the center residue is a non-binding residue. The number of binding segments in our dataset varied from 382 to 6408, and the number of non-binding segments varied from 18777 to 480307. There was an increase in the number of samples in each category compared to the Hu et al. Dataset.

**Table 1:** The statistics of the dataset using the sequence segment of length 17 for the ten metal ions.

Metal ion	Chains^a^	Binding segments	Non-binding segments
Zn^2+^	1428(142)	6408	405113
Cu^2+^	117(110)	485	33948
Fe^2+^	92(227)	382	29345
Fe^3+^	217(103)	1057	68829
Co^2+^	194(0)	875	55050
Mn^2+^	459(379)	2124	156625
Ca^2+^	1237(179)	6789	396957
Mg^2+^	1461(103)	5212	480307
K^+^	57(53)	535	18777
Na^+^	78(78)	489	27408

^a^The number of protein chains. The number in parentheses is the number of proteins in the Dataset of Hu et al.

To fairly test the performance of the proposed methods, we divided the dataset into two parts: the training dataset used to fine tune the methods by cross-validation, and the independent test dataset used to test the methods. The protein chains in the independent test accounted for 1/5 of the total data. The statistics of the two datasets are shown in Table 2.

**Table 2:** The statistics of the training dataset and the independent test dataset.

Ligand	Training dataset			Independent test dataset		
	Chains	P^a^	N^b^	Chains	P^a^	N^b^
Zn^2+^	1142	5145	321161	286	1263	83952
Cu^2+^	93	377	27548	24	108	6400
Fe^2+^	73	301	23824	19	81	5521
Fe^3+^	173	859	54945	44	198	13884
Ca^2+^	989	5256	312876	248	1533	84081
Mg^2+^	1168	4069	384365	293	1143	95942
Mn^2+^	367	1685	124543	92	439	32082
Na^+^	62	408	22411	16	81	4997
K^+^	45	410	14882	12	125	3895
Co^2+^	155	707	44300	39	168	10750

^a^The number of positive (binding) samples

^b^The number of negative (non-binding) samples.

### Methods

**Fig 1:**
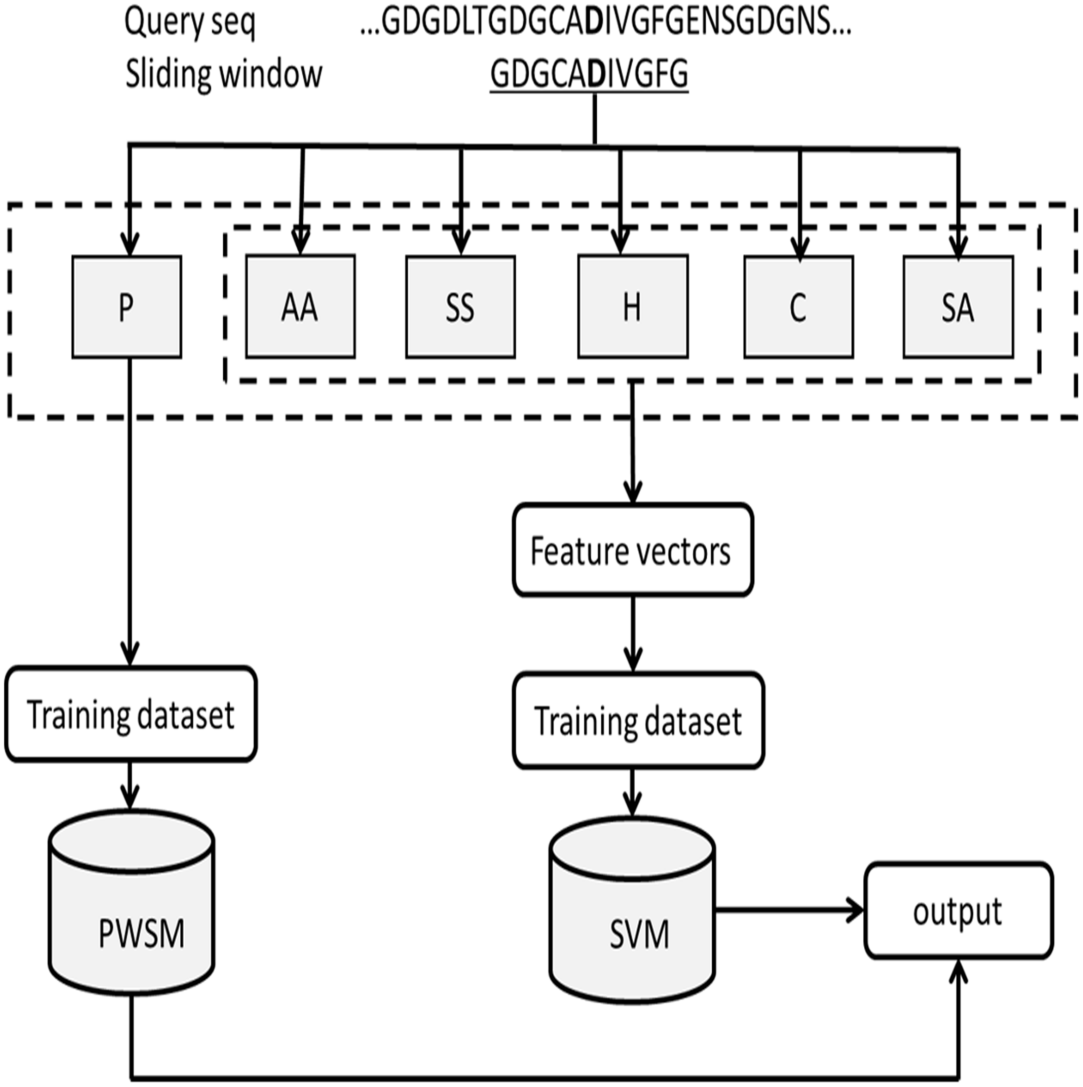
Schematic diagram of the proposed method.

This study mainly adopted the global recognition method based on the combination of the Position Weight Matrix Scoring (PWSM) algorithm and the Support Vector Machine (SVM) algorithm. First the binding sites of the ten metal ions are predicted by the PWSM algorithm using only the amino acid sequence, additional characteristic parameters are then input into the SVM to continue predicting the binding sites, and the prediction results can finally be obtained. The flowchart of this method is shown in Fig 1.

#### Position weight scoring matrix

PWSM is a classification algorithm that has been successfully used in the prediction of transcription factor binding sites in genomes and super-secondary structures [26, 27]. The scoring value is given by the following equation:

$$S=\frac{\sum_{i=1}^{L}C_{i}\left(w_{i,j}-w_{i,\text{min}}\right)}{\sum_{i=1}^{L}C_{i}\left(w_{i,\text{max}}-w_{i,\text{min}}\right)}$$ (1)

Here, $w_{i,j}=\text{log}\left(\frac{p_{i,j}}{p_{0,j}}\right),$ pi,j=ni,j+Ni21Ni+Ni

The conservation index at the i-th position may be defined by the following expression:

$$C_{i}=\frac{100}{\text{log }21}(\sum_{i=1}^{21}p_{i,j}\text{log }p_{i,j}+\text{log }21)$$ (2)

In the above equation, *w*_*i*,*j*_ is the weight probability of the j^th^ amino acids at the i^th^ position, *w*_*i*,*max*_ is the maximum value at the i^th^ position, and *w*_*i*,*min*_ is the minimum value at the i^th^ position. *L* is the length of amino acid sequence. *P*_*i*,*j*_ is the observed probability of the j^th^ amino acids at the i^th^ position, and *P*_*0*,*j*_ is background probability of the j^th^ amino acid, respectively. *N*_*i*_ is total number of all amino acids occurring at the i^th^ position, *n*_*ij*_ is the frequency of the j^th^ amino acids at the i^th^ position. The PWSM algorithm was used in this paper to extract the position conservation of amino acid residues from segments. Based on the training set, two standard position weights matrices can be constructed using the binding segments and non-binding segments, respectively. In the test set, we obtain 2 matrix scoring values for an arbitrary sequence segment using the binding and non-binding position weight matrices respectively, and the maximum value will give the segment class to which the predicted segment should belong. In addition, the two matrix scoring values can also be used as feature parameters in the SVM algorithm. For example, the position conservation of an amino acid is 21 * L dimensions for each sequence fragment, compressed into two dimensions

#### Support vector machine

The SVM is a machine learning algorithm proposed by Vapnik [28] that performs well in the classification of small samples based on the principles of structural risk minimization. We established our identification model using the Libsvm-3.21 package based on the C-SVC classifier and a radial basis function (RBF) kernel. The parameters of c and gamma were set to the default values [29]. The operation contains three steps: svm-scaling, svm-training and svm-predicting. There will be an overfitting problem in the training process when the dimensions of input vectors are too high. Thus, we reduced and refined the dimension of input vectors by using the ID algorithm PWSM algorithm to enhance the learning ability and generalization ability of the SVM.

#### The validation and evaluation metrics

We used the following four standard measures to evaluate the performance of the identification of metal ion binding residues: sensitivity (Sn), specificity (Sp), accuracy of prediction (Acc) and Matthew’s correlation coefficient (MCC). These were calculated by the following formulae:

$$S_{\text{n}}=\frac{TP}{TP+FN}\times 100\text{\%}$$ (3)

$$S_{P}=\frac{TN}{TN+FP}\times 100\text{\%}$$ (4)

$$A\text{cc}=\frac{TP+TN}{TP+TN+FP+FN}\times 100\text{\%}$$ (5)

$$MCC=\frac{\left(TP\times TN)-(FP\times FN\right)}{\sqrt{\left(TP\times FP)(TP\times FN)(TN\times FP)(TN\times FN\right)}}$$ (6)

where TP is the number of correctly identified metal ion-binding residues, TN is the number of correctly identified non-binding residues, FP is the number of non-binding residues identified as binding residues, and FN is the number of binding residues wrongly identified as non-binding residues.

The proposed method was tested by 5-fold cross-validation, which is commonly used in the prediction of ligand binding residues. The dataset was randomly divided into five sets. One set was used for testing, and the remaining four sets were used for training. This process was repeated five times in such a way that each set was used once for testing. The final performance was obtained by averaging the performances of five sets. Since the number of negative samples is much larger than that of the positive samples, to assure robustness of the proposed method, the negative samples with approximately equal numbers of positive samples were randomly extracted ten times in the 5-fold cross-validation. The final performance was obtained by averaging the performance of ten repetitions.

The training dataset was used to fine-tune the parameters of the proposed methods, and the independent test was used to test the methods.

## Results and discussion

### The study of the microenvironment and extraction of the feature parameters

In this study, we used the sliding window method to analyze the protein sequence by a fixed length *L*. The overlapping segments were generated with different window sizes (5, 7, 9, 11, 13, 15 and 17) for every protein sequence. If the central residue of the segment was a metal ion binding residue, then we assigned the segment as positive; otherwise, it was assigned as a negative segment. To generate the segment corresponding to the terminal residues in a protein sequence, we added an (L-1)/2 dummy residue "X" at both terminals of the proteins [30–34].

#### The position conservation of amino acids

**Fig 2:**
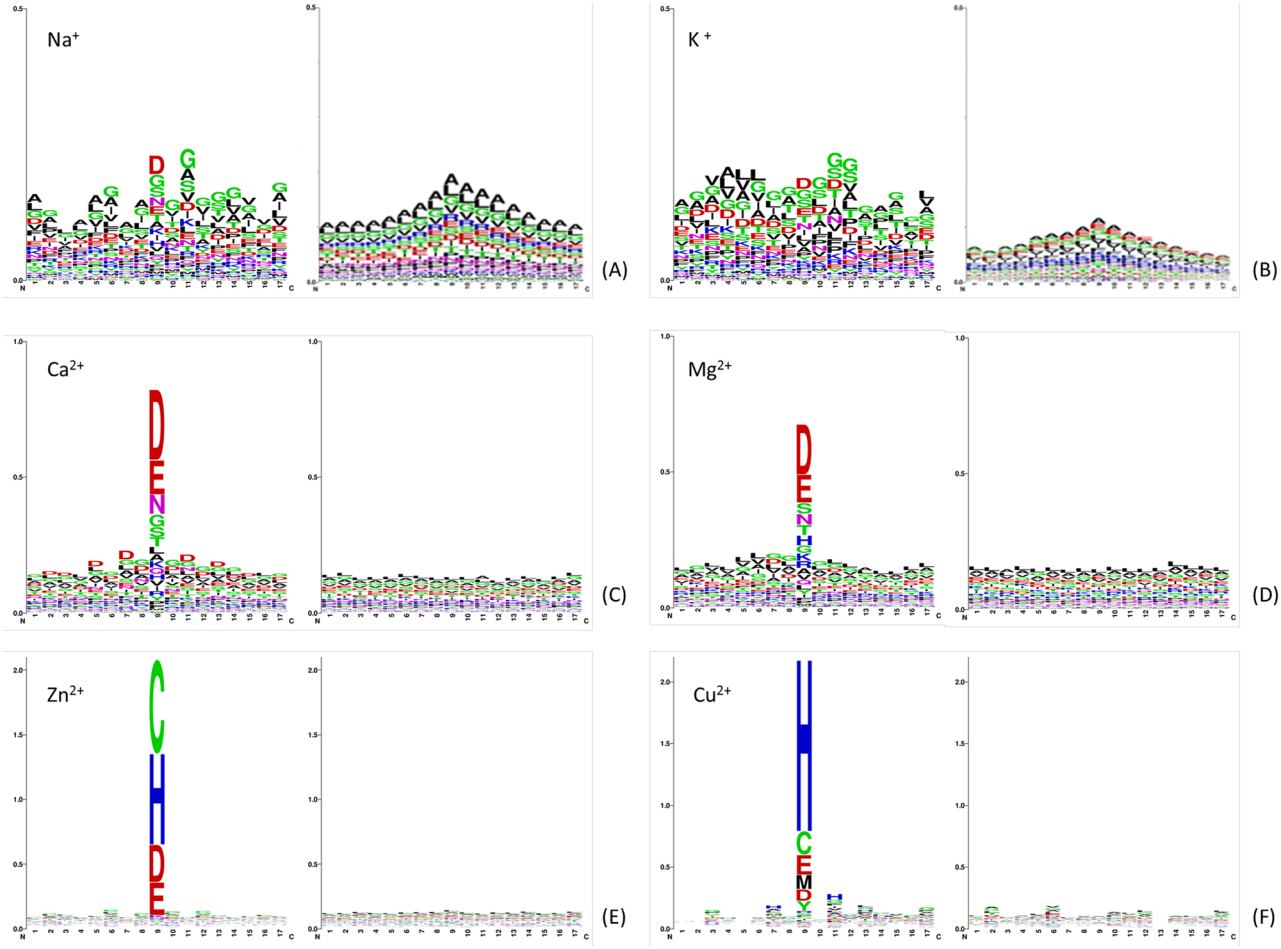
Illustration of position-specific conservation of amino acid residues in the binding and non-binding sequence segments for ions of (A) Ca^2+^, (B) Mg^2+^, (C) K^+^, (D) Na^+^, (E) Zn^2+^ and (F) Cu^2+^. The larger residues are more conserved than the smaller ones. Each subfigure of (A), (B), (C), (D), (E), and (F) contains two figures, where the left one indicates the position-specific conservation in positive sequence segments and the right one indicates the position-specific conservation in negative sequence segments.

The statistical analysis of the position conservation of 6 metal ions (the other four metal ions) was performed using WEBLOGO [35] software, and the result is shown in Fig 2 (S1 Fig). We selected a window length *L* of 17 as an example to analyze. The x-axis represents 17 positions in metal ion-binding and non-binding segments, the y-axis represents the conservation of amino acids in every position, with the height of each letter corresponding to the occurrence probability of the corresponding amino acid. As show in Fig 2, the position conservation of the alkali metal ions (Na^+^ and K^+^) binding residues and environmental residues (except the binding residues) are strong. The environmental residues of Mn^2+^ and the alkaline-earth metals (Ca^2+^, Mg^2+^) are also strong, but binding residues are stronger than those of the alkali metal ions. Interestingly, only transition metal ions (Co^2+^, Cu^2+^, Fe^2+^, Fe^3+^ and Zn^2+^) binding residues are strong, and their preferred residues are C, H, D and E amino acids. The residues of Zn^2+^ are C, H, D and E amino acids, and those of Cu^2+^ are H, C, E and D amino acids. The above analysis shows that the position conservation of amino acid residues is a good indicator of protein-metal ion binding, so it was selected as the feature information to further develop an effective identification model.

#### The amino acid composition

**Fig 3:**
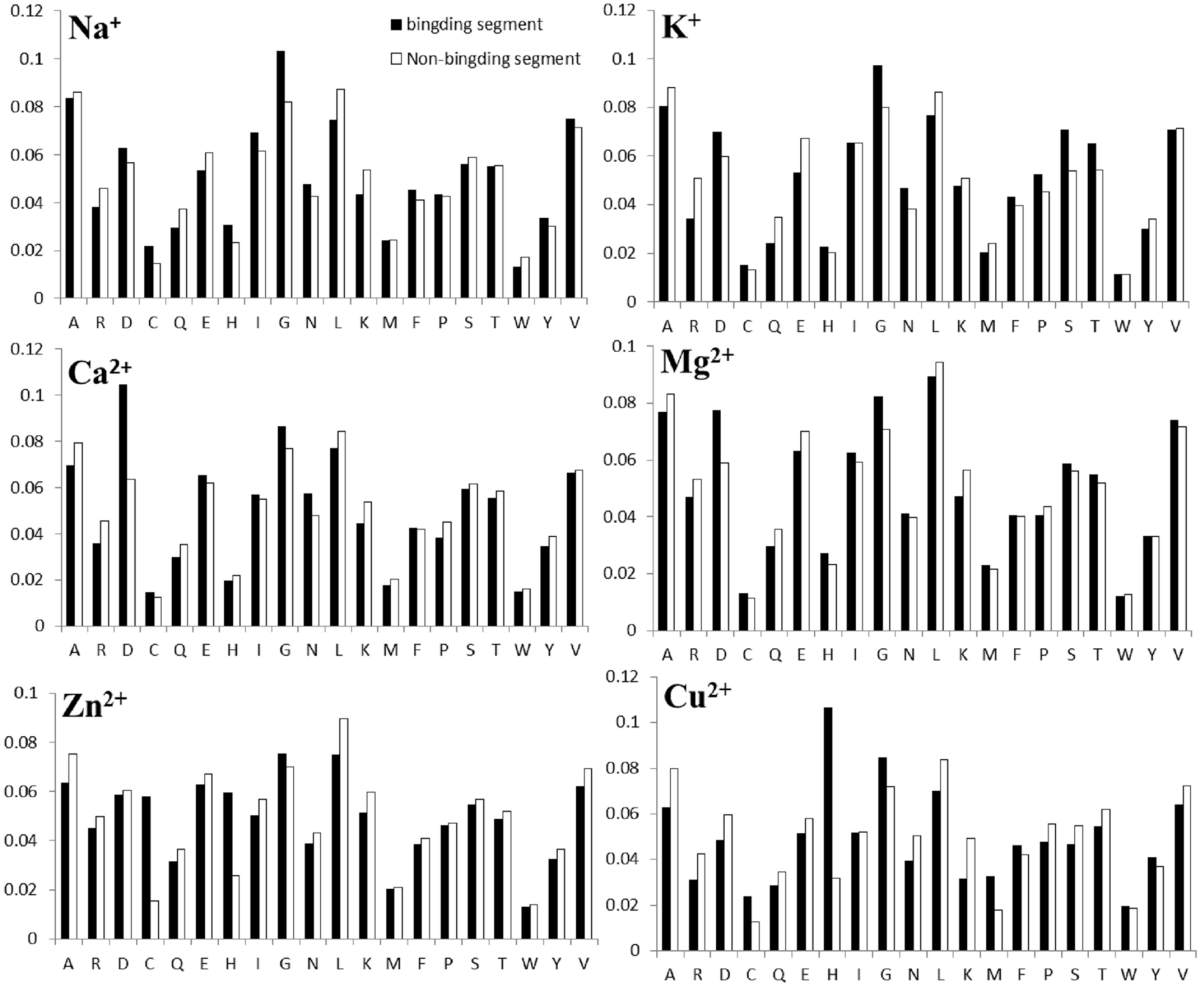
Statistical analysis of the amino acid composition in positive and negative segments for Na^+^, K^+^, Mg^2+^, Ca^2+^, Zn^2+^, and Cu^2+^.

The amino acid composition as important feature information is commonly used in the identification of ligand binding residues and other studies [32, 36]. Therefore, we analyzed the amino acid composition in metal ion-binding segments and non-binding segments of six metal ions (the other four metal ions). As shown in Fig 3 (S2 Fig), the x-axis represents 20 amino acids in metal ion-binding and non-binding segments, and the y-axis represents the occurrence probability of amino acids in every segment. There was a significant difference between binding and non-binding segments; residues D and G had larger occurrence in non-binding segments than in non-binding segments. In addition, although glutamic acid E is a preferred residue (Fig 2), there are more E residues in non-binding fragments (Fig 3). This reflects the fact that the distribution of the amino acids and the amino acid composition information are not the same. Thus, the amino acid composition was also selected as feature information.

In this study, the amino acid composition was reduced and refined by the Increment of Diversity (ID) algorithm, a classifier that has been successfully used in the identification of protein folds and subcellular localization [37, 38] in recent years. In the state space of dimension *S*, for a vector *X*: [*n*_*1*_, *n*_*2*_, …, *n*_*s*_] the measure of diversity source was

$$D(X)=N\text{log }N-\sum_{i=1}^{s}n_{i}\text{log }n_{i}.$$ (7)

For two state spaces of dimension *S*, for vectors *X*: [*n*_*1*_, *n*_*2*_, …, *n*_*s*_] and *Y*: [*m*_*1*_, *m*_*2*_, …, *m*_*s*_], the measure of mixed diversity source *X*+*Y* was

$$D(X,Y)=(N+M)\text{log }b(N+M)-\sum_{i=1}^{s}\left(ni+mi)\text{log }b(ni+mi\right).$$

The increment of diversity between the source of diversity *X* and *Y* was

ID(X,Y)=D(X+Y)−D(X)−D(Y) (8)

The component information was input into the ID algorithm. The standard discrete source is constructed by training. Two discrete increment (ID) values can be obtained for each segment of the test set. Finally, the obtained two-dimensional ID value is taken as the characteristic parameter input to the SVM algorithm. Thus, the frequencies of 21 amino acids (including dummy amino acids X) of each sequence fragment is a 21-dimensional vector compressed into two dimensions.

#### Physicochemical properties of amino acids

Amino acids have different physicochemical characteristics from their side chains. The interaction between the ligand binding residues and metal ions are probabilistic in that the metal ions prefer to bind with specific side-groups of residues. It was thus important to extract information from the side chains. Amino acids can be grouped into different categories according to different criteria [39, 40]. Here, we extracted the information of hydrophilicity and hydrophobicity (H) and polarization charge (C) as feature parameters. The 20 amino acids are grouped into 6 kinds according to hydrophilicity and hydrophobicity (Table 3) and three kinds according to polarization charge (Table 4) [41, 42].

**Table 3:** Hydrophilic-hydrophobic classification of amino acids.

Classification	Amino Acids	Classification	Amino Acids
strongly hydrophilic	R, D, E, N, Q, K, H	Proline	P
weakly hydrophilic	L, I, V, A, M, F	Glycine	G
strongly hydrophobic	S, T, Y, W	Cysteine	C

**Table 4:** The polarization charge property of amino acids.

Classification	Amino Acids
positive charged	K, R, P
negative charged	D, E
uncharged	N, Q, H, L, I, V, A, M, F, S, T, Y, W, C, G

#### Secondary structure and solvent accessibility information

The prediction of secondary structure and solvent accessibility is a key step in moving from the sequence to the tertiary structure of proteins, reflecting spatial structure information of the backbone and side chains, respectively [43]. In this study, secondary structure and solvent accessibility information were predicted using ANGLOR [44] software. We counted frequencies of three secondary structure types (alpha-helix (H), beta-strand (E) and coil(C)), using PWSM to extract the position conservation of secondary structure. The relative solvent accessibility (SA) is generally represented as a Boolean value denoting whether the residue is buried (RSA < 25%) or exposed (RSA > 25%). In this study, we did not adopt the above threshold Boolean value directly. Instead, the distribution of the relative solvent accessibility for binding and non-binding residues was performed, and then, appropriate thresholds were chosen to categorize the relative solvent accessibilities into different groups.

**Fig 4:**
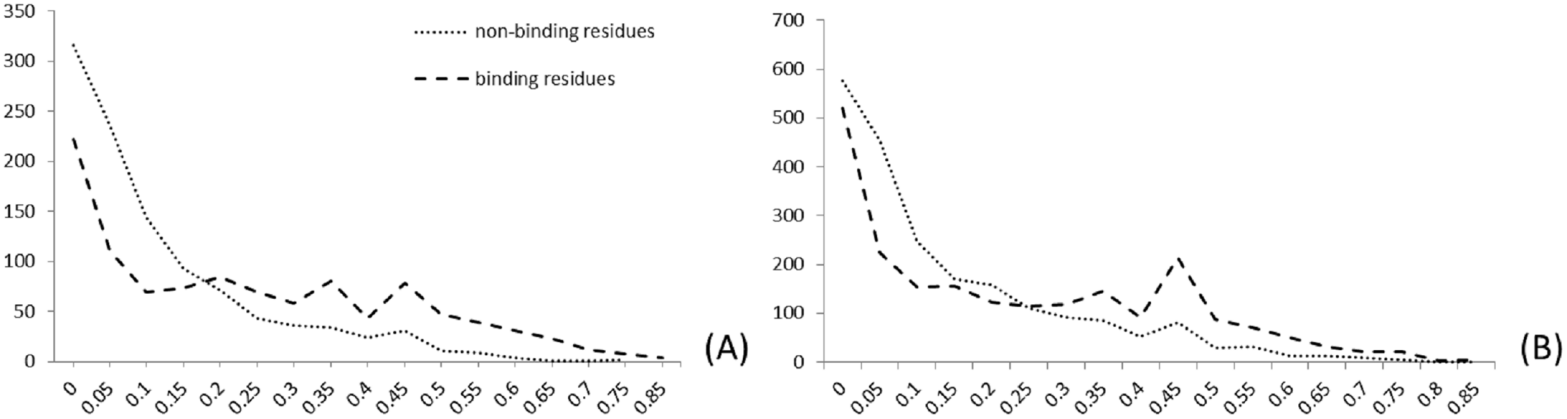
The distribution of relative solvent accessibilities for binding and non-binding residues of (A) Fe^3+^ ligand and (B) Mn^2+^ ligand.

Fig 4 shows the example distribution of Fe^3+^ and Mn^2+^ ligands. As seen from subfigure (A), there is an intersection at 0.25, and there are peaks at 0.35 and 0.45. Similarly, there is a peak at 0.25 and 0.45 in subfigure (B). Based on these observations, several partitions were evaluated. The experiments showed that the following partition yielded the best results. The relative solvent accessibilities are mainly concentrated in four regions (0~0.2, 0.2~0.45, 0.45~0.6 and 0.6~0.85) represented by 4 letters (I, J, M, and N):

$$g(x)=\{\begin{array}{cc} I & x\in (0,0.2]\\ J & x\in (0.2,0.45]\\ M & x\in (0.45,0.6]\\ N & x\in (0.6,0.85] \end{array}$$ (9)

Here, we refined the solvent accessibility information via the PWSM algorithm, extracting the two-dimensional matrix scoring as the characteristic parameter of the SVM algorithm.

### The optimal window length

The binding sites for ten metal ions were extracted from the BioLiP database, containing the alkali metals (Na^+^ and K^+^), the alkaline-earth metals (Ca^2+^ and Mg^2+^), and the transition metals (Zn^2+^, Cu^2+^, Fe^2+^, Fe^3+^, Co^2+^ and Mn^2+^). The optimal window lengths of the sequence segments for different metal ions were determined from the results of the statistical analysis of the amino acid position conservation. If there were obvious differences between positive and negative segments of conservation information, the optimal window lengths could be determined directly. Otherwise, we computed and analyzed seven windows (L = 5, 7, 9, 11, 13, 15, and 17), combined with the four standard measures (Sp, Sn, Acc, and MCC) to obtain the optimal window. Our selected optimal windows (see Table 5) for the ten metal ions varied from 7 to 13, which was smaller than that of ATP and NAD [32,33] ligands (17 in general). Since the volume of metal ions is generally small, they usually only bind with a few residues. Thus, the optimal window length of metal ions should be smaller than that of the larger ligands. Our selection of window length fits the interacting mechanisms of protein-ligands.

**Table 5:** Performance of PWSM by 5-fold cross-validation.

Ligand	Optimal windows (W)	Sn (%)	Sp (%)	Acc (%)	MCC
Zn^2+^	7	**94.8**	**83.5**	**89.2**	**0.788**
Cu^2+^	13	**85.6**	**91.3**	**88.5**	**0.770**
Fe^2+^	9	**92.7**	**78.0**	**85.3**	**0.715**
Fe^3+^	9	**86.7**	**78.1**	**82.4**	**0.650**
Co^2+^	11	**74.5**	**85.3**	**79.9**	**0.601**
Mn^2+^	7	87.3	63.6	75.9	0.526
Ca^2+^	9	57.9	80.6	69.2	0.395
Mg^2+^	9	55.6	80.9	68.3	0.378
K^+^	11	61.3	72.0	66.6	0.335
Na^+^	9	30.1	95.3	62.7	0.335

### Identification of the binding residues for ten metal ions by PWSM

We identified the metal ion binding residues using position amino acid conservation as the feature parameter via the PWSM algorithm. As shown in Table 5, the metal ions Zn^2+^, Cu^2+^, Fe^2+^, Fe^3+^, and Co^2+^ yielded satisfactory results with Acc percentages greater than 79.9% and MCC values greater than 0.6. These metal ions were sensitive to the position amino acid conservation and could be identified by the PWSM algorithm. Ca^2+^, Mg^2+^, Mn^2+^, Na^+^ and K^+^ had various preferred residues and were less sensitive to the position amino acid conservation. The results for these metal ions were less accurate, probably because there were not enough features. In the next section, extra feature parameters have been extracted to further enhance the performance.

### Identification of binding residues for the metal ions by SVM

#### Five-fold cross-validation results

To improve the prediction performance for Ca^2+^, Mg^2+^, Mn^2+^, Na^+^ and K^+^, the dimensions of amino acid composition were reduced and refined using the ID algorithm. The obtained ID (AA) values were combined with position conservation values (S(P)) calculated by the PWSM and input to the SVM. The results of 5-fold cross-validation are given in Table 6. As seen, the performance has been significantly improved. For example, the Sn value of Na^+^ has been significantly improved from 30.1% to 73.6%.

**Table 6:** The performance of SVM(S(P)+ID(AA)) by 5-fold cross-validation.

Ligand	Sn (%)	Sp (%)	Acc (%)	MCC
Mn^2+^	73.4	83.9	78.7	0.577
Ca^2+^	71.1	58.0	70.8	0.422
Mg^2+^	64.2	73.9	69.0	0.382
K^+^	72.2	67.5	69.8	0.397
Na^+^	73.6	70.1	71.9	0.438

We sequentially added to the feature parameter from the PWSM the values of secondary structure (S(SS)), hydrophobicity and hydrophilicity (S(H)), polarization charge (S(C)) and solvent accessibility information (S(SA)). The performance was stably improved by the additions. Table 7 lists the results for K^+^ (the results for other ions are provided in the supporting information). The Acc value was improved from 66.6% to 80.3%; the MCC value was increased from 0.335 to 0.607; and the values of Sp and Sn were balanced between 77.3% and 83.2%, respectively.

**Table 7:** Recognition results of ligand binding residues for K^+^ ion.

Algorithm (Parameter)	Sn (%)	Sp (%)	Acc (%)	MCC
PWSM(P)	61.3	72.0	66.6	0.335
SVM(ID(AA)+S(P))	72.2	67.5	69.8	0.397
SVM(ID(AA)+S(P)+SS+S(SS))	74.2	67.3	70.7	0.416
SVM(ID(AA)+S(P)+SS+S(SS)+S(H))	78.5	72.7	75.6	0.513
SVM(ID(AA)+S(P)+SS+S(SS)+S(H)+S(C))	70.2	88.1	79.2	0.593
SVM(ID(AA)+S(P)+SS+S(SS)+S(H)+S(C)+S(SA))	77.3	83.2	80.3	0.607

ID(AA) represents the ID values of amino acid composition, S(P) represents the scoring values of position amino acid conservation information, SS represents the scoring values of the frequency of secondary structure, S(SS) represents the scoring values of second structure information, and S(H) represents the scoring values of hydrophobicity and hydrophilicity information. S(C) represents the scoring values of polarization charge information. S(SA) represents the scoring values of solvent accessibility information.

We found that the results were not further improved by adding S(H) to the Ca^2+^ model or by adding S(C) to Mn^2+^ (see Table 8). We discarded S(H) in Ca^2+^ and the corresponding values of Acc and MCC declined, which demonstrated that Ca^2+^ binding residues were not sensitive to the single S(H) parameter, but this feature is significant in the calcium ion‘s model as a whole (see Table 8). We also discarded S(C) in Mn^2+^, as the values of Acc and MCC were almost unchanged. Mn^2+^ binding residues thus were not sensitive to S(C) as a whole (see lines 1 to 4 of Mn^2+^ in Table 8).

**Table 8:** The performance of Ca^2+^ and Mn^2^+ by 5-fold cross-validation with feature tuning.

ID	Algorithm (Parameter)	Sn (%)	Sp (%)	Acc (%)	MCC
Ca^2+^	SVM(ID(AA)+S(P)+SS+S(SS))	69.0	75.7	72.3	0.448
	SVM(ID(AA)+S(P)+SS+S(SS)+S(H))	68.3	76.5	72.4	0.450
	SVM(ID(AA)+S(P)+SS+S(SS)+S(H)+S(C)+S(SA))	69.7	82.0	75.8	0.521
	SVM(ID(AA)+S(P)+SS+S(SS)+S(C)+S(SA))	71.3	79.1	74.8	0.502
Mn^2+^	SVM(ID(AA)+S(P)+SS+S(SS)+S(H))	77.6	84.2	80.8	0.618
	SVM(ID(AA)+S(P)+SS+S(SS)+S(H)+S(C))	78.2	83.9	81.1	0.622
	SVM(ID(AA)+S(P)+SS+S(SS)+S(H)+S(C)+S(SA))	82.1	84.4	83.2	0.664
	SVM(ID(AA)+S(P)+SS+S(SS)+S(H)+S(SA))	82.0	84.8	83.4	0.667

The final results of the SVM algorithm for identifying the binding residues of the ten metal ions are listed in Table 9. As seen, the Acc values are greater than 74.8%, and the MCC values are greater than 0.502 for all ions; Zn^2+^ is the highest, with an Acc value of 99.7% and an MCC value of 0.993. This may be because large zinc finger domains exist in zinc proteins where more than 90% of the Zn^2+^ preferred residues are C, H, D, and E. Both Zn^2+^ and Ca^2+^ are abundant within the cell and have more known binding sites. Zn^2+^ typically utilizes fewer ligands, with more side chains, and has fewer known sites, while Ca^2+^ utilizes both side-chain and main-chain ligands, uses more ligands, and has more binding sites. According to our previous work [23], the average numbers of binding residues per ligand for Zn^2+^ and Ca^2+^ are 3.4 and 4.4, respectively. The performance of Ca^2+^ was much lower than that of Zn^2+^, which may be caused by the complicated binding mechanism of Ca^2+^. The preferred residues and microenvironment of Ca^2+^ were influenced by multiple feature parameters. For example, after adding the feature parameter of PWSM values of secondary structure(S(SS)) the result was improved, which supported the observation that backbone carbonyl oxygens, rather than side-chain oxygens, frequently bind with Ca^2+^ [22]. The average numbers of binding residues per ligand for Na^+^ and K^+^ were 5.4 and 6.5, respectively, both of which also had a lower performance in comparison with Zn^2+^. This phenomenon indicates that the greater the average number of binding residues, the more complicated the binding mechanism.

**Table 9:** The performance of the metal-ion-binding-residue prediction of SVM using 5-fold cross-validation.

Ligand	Sn (%)	Sp (%)	Acc (%)	MCC
Zn^2+^	99.8	99.5	99.7	0.993
Cu^2+^	95.5	97.1	96.3	0.926
Fe^2+^	91.9	90.7	91.3	0.826
Fe^3+^	86.9	88.7	87.8	0.756
Ca^2+^	71.3	79.1	74.8	0.502
Mg^2+^	76.6	73.9	75.3	0.505
Mn^2+^	82.1	84.4	83.2	0.664
Na^+^	82.2	76.2	79.4	0.586
K^+^	77.3	83.2	80.3	0.607
Co^2+^	80.8	85.1	83.0	0.660

#### Independent test results

The proposed method was tested on the independent test set and compared with the IonSeq method, which is a recently developed sequence-based method. The performance of the proposed method was obtained by independent testing, while the performance of the IonSeq method was taken directly from a paper in which it was obtained by cross-validation. The results are shown in Table 10. The results for Zn^2+^ are relatively high; the results for Na^+^ and K^+^ are relatively low, and the prediction trend for different metal ions is consistent with that obtained using IonSeq [23]. Since the number of non-binding residues is far greater than that of binding residues, the results are lower than those obtained by cross-validation. All the MCC values of the proposed method are slightly lower than those from IonSeq. There are three possible reasons for this result. First, the advantage of the proposed method is that it has a higher recognition accuracy at the fragment level, and the results from the IonSeq method are directly taken from the original paper; those results were obtained by cross-validation, while the results of our method were calculated by independent testing. Second, the datasets used by the methods are slightly different. Although both datasets were derived from the BioLiP database, the number of samples in this paper is much larger than that of the dataset used for IonSeq (Table 1). Third, the IonSeq program uses the Adaboost algorithm [45] to construct the SVM model, which is aimed at the prediction of the protein chain in the real case; in this paper we constructed a model mainly for the prediction of the fragment. Additionally, the solvent accessibility partition in this paper is not yet accurate enough, and we will continue to improve it in further research. The two methods thus have their own advantages and can only be roughly compared.

**Table 10:** Comparison of our independent test results with IonSeq.

Ligand	L	Method	Sn (%)	Sp (%)	Acc (%)	MCC
Zn^2+^	13	IonSeq	43.56	99.75	99.21	0.5043
	7	OUR’S	94.1	84.3	84.4	0.2528
Cu^2+^	15	IonSeq	50.65	99.69	99.01	0.5772
	13	OUR’S	91.7	82.9	83.0	0.2458
Fe^2+^	9	IonSeq	54.08	99.51	98.84	0.6370
	9	OUR’S	90.1	73.6	73.9	0.1708
Fe^3+^	11	IonSeq	52.27	99.81	99.21	0.2111
	9	OUR’S	87.9	72.7	72.9	0.1584
Ca^2+^	9	IonSeq	22.72	99.04	98.18	0.1825
	9	OUR’S	59,5	79.2	78.9	0.1251
Mg^2+^	15	IonSeq	5.57	99.98	99.49	0.4553
	9	OUR’S	50.2	81.9	81.6	0.0871
Mn^2+^	11	IonSeq	31.07	99.82	99.01	0.1516
	7	OUR’S	76.5	79.8	79.8	0.1599
Na^+^	13	IonSeq	77.14	74.04	74.09	0.2283
	9	OUR’S	33.3	78.2	77.5	0.0348
K^+^	11	IonSeq	8.52	99.88	97.32	0.2283
	11	OUR’S	45.6	62.8	62.3	0.0301
Co^2+^	-	IonSeq	-	-	-	-
	11	OUR’S	0.732	0.823	0.822	0.176

## Conclusion

In this study we proposed effective methods for predicting the binding residues of ten metal ions. The following conclusions may be drawn. (1) The optimal window lengths of metal ions were shorter than those of ligands with a larger volume. (2) The metal ions Co^2+^, Cu^2+^, Fe^2+^, Fe^3+^ and Zn^2+^ were sensitive to amino acid position information and could be identified by the PWSM. (3) The metal ions Mn^2+^, Na^+^, K^+^, Ca^2+^ and Mg^2+^ were influenced by multiple feature parameters including the ID of amino acid composition, second structure (SS), hydrophobicity and hydrophilicity (H), polarization charge (C) and solvent accessibility (SA). After adding these feature parameters to the SVM, the identification results were significantly improved. (4) The binding residues of Ca^2+^ were not sensitive to the single S(H) parameter. The binding residues of Mn^2+^ were not sensitive to the S(C) parameter. In future work, we will try to add 3D structure information to identify metal ion binding residues and improve the predicted results.

## Supporting information

S1 Fig Illustration of position conservation of amino acid residues in the binding and non-binding segments for (A) Fe^3+^, (B) Fe^2+^, (C) Co^2+^ and (D) Mn^2+^. (DOCX)

S2 Fig Statistical analysis of amino acid composition in positive and negative segments for Fe^2+^, Fe^3+^, Co^2+^, and Mn^2+^. (DOCX)

S1 Table Recognition results of Ca^2+^ ligand binding residues. (DOCX)

S2 Table Recognition results of Mg^2+^ ligand binding residues. (DOCX)

S3 Table Recognition results of Mn^2+^ ligand binding residues. (DOCX)

S4 Table Recognition results of Na^+^ ligand binding residues. (DOCX)
