# Supplementary material for: Identification of metal ion binding sites based on amino acid sequences
Source: PLoS One. 2017 Aug 30;12(8):e0183756. doi: 10.1371/journal.pone.0183756 (PMC5576659; doi:10.1371/journal.pone.0183756)
Supplement: S1 Fig — (DOCX) [file pone.0183756.s001.docx]

**S1 Fig. Illustration of position conservation of amino acid residues in the binding and non-binding segments for (A) Fe^3+^, (B) Fe^2+^, (C)Co^2+^ and (D)Mn^2+^**

**^
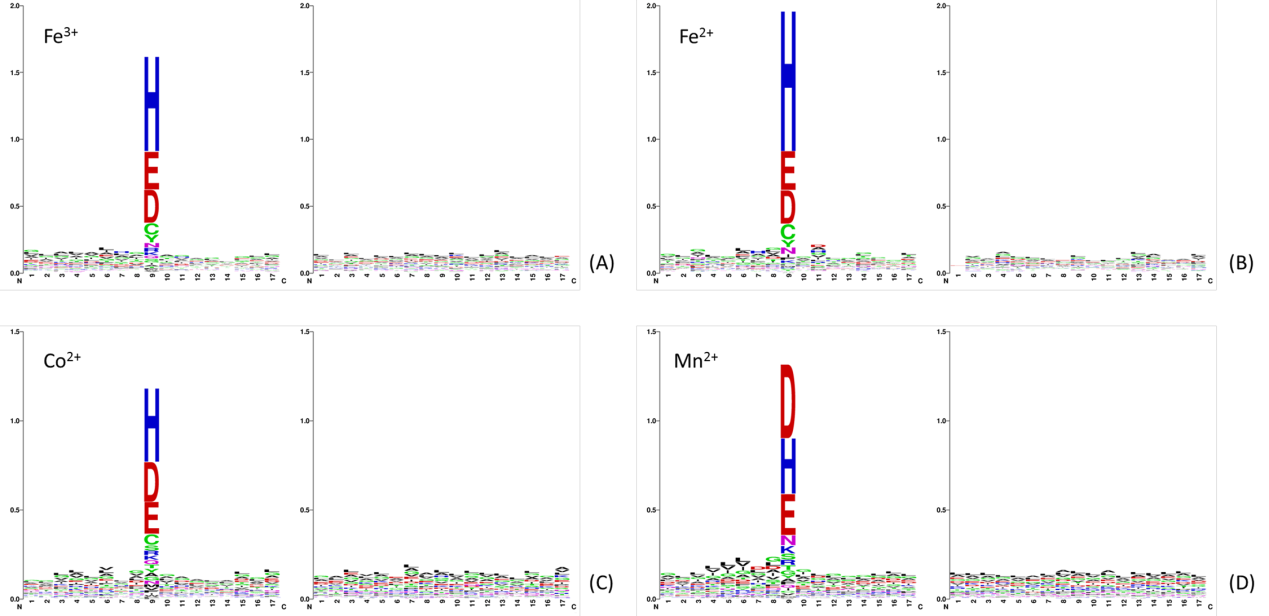
^**
