# Supplementary material for: Identification of metal ion binding sites based on amino acid sequences
Source: PLoS One. 2017 Aug 30;12(8):e0183756. doi: 10.1371/journal.pone.0183756 (PMC5576659; doi:10.1371/journal.pone.0183756)
Supplement: S2 Fig — (DOCX) [file pone.0183756.s002.docx]

**S2 Fig. Statistical analysis of amino acid composition in positive and negative segments for Fe^3+^, Fe^2+^,Co^2+^, and Mn^2+^**

**
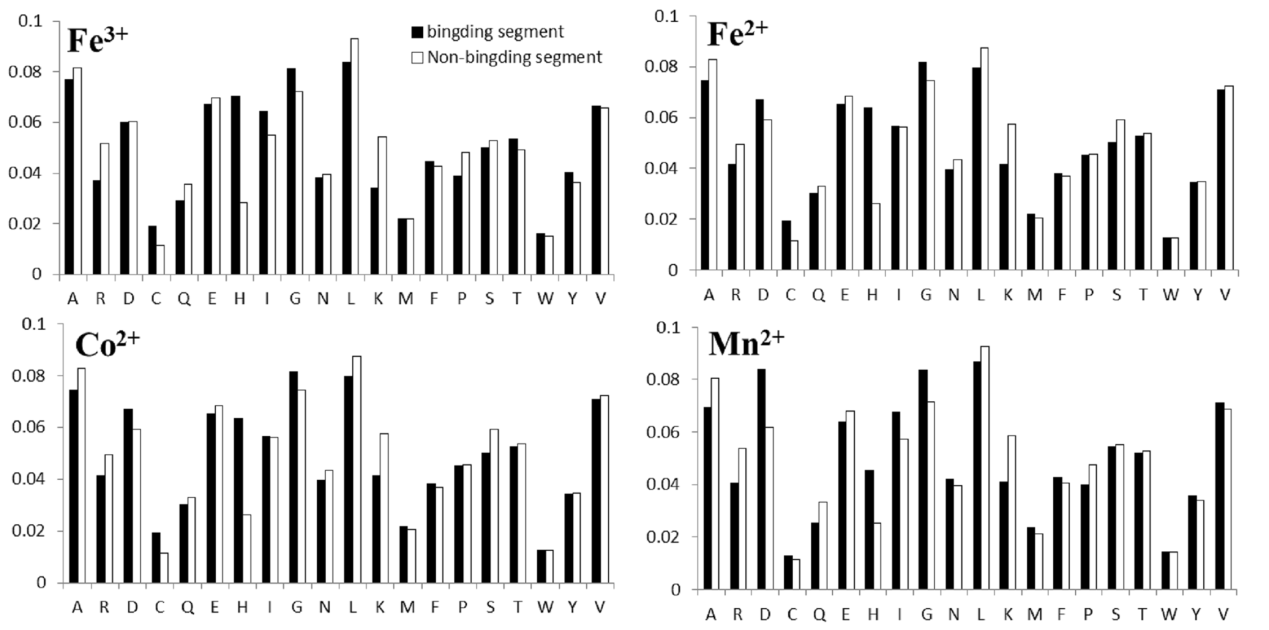
**
