# Supplementary material for: Identification of metal ion binding sites based on amino acid sequences
Source: PLoS One. 2017 Aug 30;12(8):e0183756. doi: 10.1371/journal.pone.0183756 (PMC5576659; doi:10.1371/journal.pone.0183756)
Supplement: S1 Table — (DOCX) [file pone.0183756.s003.docx]

**S1 Table. Recognition results of Ca^2+^ ligand binding residues**

| Algorithm(Parameter) | Sp | Sn | ACC | MCC |
| --- | --- | --- | --- | --- |
| PWSM(P) | 57.9% | 80.6% | 69.2% | 0.395 |
| SVM(ID(AA)+S(P)) | 62.5% | 79.2% | 70.8% | 0.422 |
| SVM(ID(AA)+S(P)+SS+S(SS)) | 69.0% | 75.7% | 72.3% | 0.448 |
| SVM(ID(AA)+S(P)+SS+S(SS)+S(H)) | 68.3% | 76.5% | 72.4% | 0.450 |
| SVM(ID(AA)+S(P)+SS+S(SS)+S(H)+S(C)) | 74.2% | 75.3% | 74.7% | 0.495 |
| SVM(ID(AA)+S(P)+SS+S(SS)+S(H)+S(C)+S(SA)) | 69.7% | 82.0% | 75.8% | 0.521 |
