# Supplementary material for: Identification of metal ion binding sites based on amino acid sequences
Source: PLoS One. 2017 Aug 30;12(8):e0183756. doi: 10.1371/journal.pone.0183756 (PMC5576659; doi:10.1371/journal.pone.0183756)
Supplement: S2 Table — (DOCX) [file pone.0183756.s004.docx]

**S2 Table. Recognition results of Mg^2+^ ligand binding residues**

| Algorithm(Parameter) | Sp | Sn | ACC | MCC |
| --- | --- | --- | --- | --- |
| PWSM(P) | 55.6% | 80.9% | 68.3% | 0.378 |
| SVM(ID(AA)+S(P)) | 64.2% | 73.9% | 69.0% | 0.382 |
| SVM(ID(AA)+S(P)+SS+S(SS)) | 67.1% | 71.8% | 69.4% | 0.389 |
| SVM(ID(AA)+S(P)+SS+S(SS)+S(H)) | 72.5% | 70.5% | 71.5% | 0.430 |
| SVM(ID(AA)+S(P)+SS+S(SS)+S(H)+S(C)) | 71.8% | 74.5% | 73.1% | 0.462 |
| SVM(ID(AA)+S(P)+SS+S(SS)+S(H)+S(C)+S(SA)) | 76.6% | 73.9% | 75.3% | 0.505 |
| SVM(ID(AA)+S(P)+S(H)+S(C)+S(SA)) | 70.1% | 76.5% | 72.9% | 0.462 |
