# Supplementary material for: Identification of metal ion binding sites based on amino acid sequences
Source: PLoS One. 2017 Aug 30;12(8):e0183756. doi: 10.1371/journal.pone.0183756 (PMC5576659; doi:10.1371/journal.pone.0183756)
Supplement: S3 Table — (DOCX) [file pone.0183756.s005.docx]

**S3 Table. Recognition results of Mn^2+^ ligand binding residues**

| Algorithm(Parameter) | Sp | Sn | ACC | MCC |
| --- | --- | --- | --- | --- |
| PWSM(P) | 88.0% | 60.5% | 74.7% | 0.507 |
| SVM(ID(AA)+S(P)) | 73.9% | 84.1% | 79.0% | 0.583 |
| SVM(ID(AA)+S(P)+SS+S(SS)) | 78.1% | 82.7% | 80.4% | 0.608 |
| SVM(ID(AA)+S(P)+SS+S(SS)+S(H)) | 77.6% | 84.2% | 80.8% | 0.618 |
| SVM(ID(AA)+S(P)+SS+S(SS)+S(H)+S(C)) | 78.2% | 83.9% | 81.1% | 0.622 |
| SVM(ID(AA)+S(P)+SS+S(SS)+S(H)+S(C)+S(SA)) | 82.1% | 84.4% | 83.2% | 0.664 |
