# Supplementary material for: Identification of metal ion binding sites based on amino acid sequences
Source: PLoS One. 2017 Aug 30;12(8):e0183756. doi: 10.1371/journal.pone.0183756 (PMC5576659; doi:10.1371/journal.pone.0183756)
Supplement: S4 Table — (DOCX) [file pone.0183756.s006.docx]

**S4 Table. Recognition results of Na^+^ ligand binding residues**

| Algorithm(Parameter) | Sp | Sn | ACC | MCC |
| --- | --- | --- | --- | --- |
| PWSM(P) | 30.1% | 95.3% | 62.7% | 0.335 |
| SVM(ID(AA)+S(P)) | 73.6% | 70.1% | 71.9% | 0.438 |
| SVM(ID(AA)+S(P)+SS+S(SS)) | 73.2% | 72.6% | 72.9% | 0.458 |
| SVM(ID(AA)+S(P)+SS+S(SS)+S(H)) | 78.0% | 75.9% | 76.8% | 0.535 |
| SVM(ID(AA)+S(P)+SS+S(SS)+S(H)+S(C)) | 77.3% | 78.2% | 77.7% | 0.552 |
| SVM(ID(AA)+S(P)+SS+S(SS)+S(H)+S(C)+S(SA)) | 82.2% | 76.2% | 79.4% | 0.586 |
